# MicroRNA warfare: how chickens combat *Klebsiella variicola* with gga-miR-2954

**DOI:** 10.3389/fcimb.2025.1544506

**Published:** 2025-05-08

**Authors:** Lei Yin, Xuehuai Shen, Dongdong Yin, Hongyan Hou, Jieru Wang, Ruihong Zhao, Kezong Qi, Yin Dai, Xiaocheng Pan

**Affiliations:** ^1^ Livestock and Poultry Epidemic Diseases Research Center of Anhui Province, Institute of Animal Husbandry and Veterinary Science, Anhui Academy of Agricultural Sciences, Hefei, Anhui, China; ^2^ Anhui Province Key Laboratory of Livestock and Poultry Product Safety, Institute of Animal Husbandry and Veterinary Science, Anhui Academy of Agricultural Sciences, Hefei, Anhui, China; ^3^ Anhui Province Key Laboratory of Veterinary Pathobiology and Disease Control, College of Animal Science and Technology, Anhui Agricultural University, Hefei, China

**Keywords:** Klebsiella variicola, spleen, miRNAs, immune response, inflammation

## Abstract

*Klebsiella variicola* is a member of *Klebsiella pneumoniae* complex and an emerging zoonotic pathogen. As part of the lymphatic system, the spleen plays a pivotal role in destroying invading pathogens. Various microRNAs (miRNAs) are involved in host resistance to pathogens. However, specific miRNAs that act against *K. variicola* remain unknown. Therefore, RNA sequencing (RNA-Seq) of the miRNA profile of the chicken spleen was conducted to further clarify the host immune response to *K. variicola* infection. Challenge of 7-day-old chicks with *K. variicola* strain AHKV-S01 caused severe damage and enlargement of the spleen. In total, 22 differentially expressed (DE) miRNAs (fold change>2, *q*< 0.05) were identified. Functional annotation analysis of the target genes of DE miRNAs found that signaling pathways related to innate immunity, inflammation, and metabolism were significantly enriched. Notably, expression of gga-miR-2954 was significantly upregulated in the infection group as compared to the control group. *In vitro*, gga-miR-2954 directly repressed luciferase reporter gene activity by binding to the 3′ untranslated regions of *STAB1*. Overexpression of gga-miR-2954 in HD11 macrophages significantly inhibited expression of *STAB1*, which is involved in activation of several proinflammatory cytokines. *K. variicola* induced damage to the spleen by over activation of inflammatory and innate immune responses. The observed changes to the miRNA expression profile of the chicken spleen elucidate host immune responses to *K. variicola* infection, providing critical insights for developing novel therapeutic strategies to enhance chicken resistance against this pathogen.

## Introduction

The *Klebsiella pneumoniae* complex consists of seven *K. pneumoniae*-related species, including *K. variicola*, which is capable of infecting plants, insects, animals, and humans (Li et al., 2024). Moreover, *K*. *variicola* is considered an emerging pathogen worldwide and is often more virulent than *K*. *pneumonia* (Rodríguez-Medina et al., 2019). The capsule serves as a core virulence factor in *Klebsiella*, with its synthesis dynamically regulated by environmental cues (e.g., osmolarity, temperature) and host immune pressure. Studies demonstrate that capsular polysaccharide synthesis (CPS) genes are highly expressed during early infection stages to evade immune clearance, while their expression is epigenetically downregulated in later colonization phases through promoter-region IS element insertions or methylation, thereby enhancing biofilm formation (Ernst et al., 2020; Song et al., 2024). Additionally, pilus expression exhibits tissue specificity: Type 1 pili, mediated by the fim gene cluster, facilitate epithelial adhesion during intestinal colonization, whereas Type 3 pili (mrk gene cluster) dominate endothelial invasion and immune evasion in systemic infections (Fan et al., 2023). *Klebsiella* orchestrates virulence factor expression to navigate host immune pressure, achieving multifaceted adaptation across diverse host environments. Pan-genome phylogenetic analyses showed that *K*. *variicola* isolated from diseased chickens was evolutionarily closely related to the human *K*. *variicola* strain X39 (Shen et al., 2021). Thus, avian strains of *K*. *variicola* could be pathogenic to other animals and even humans. In addition, *K*. *variicola* tends to be highly resistant to multiple antimicrobials, thereby demonstrating potential to complicate treatment (Ge et al., 2023). Therefore, to reduce economic losses to the poultry industry and to protect animal and human health, it is critical to elucidate the mechanisms underlying host resistance and immune responses against *K*. *variicola* infection.

MicroRNAs (miRNAs) are a class of small non-coding RNAs of 20–23 nucleotides that play key regulatory roles in host–pathogen interactions by repressing or degrading target mRNAs at the post-transcriptional level (Chen et al., 2024). Notably, a recent study implicated miR-92a, TLR4, and various cytokines in the response of bovine mammary epithelial cells to infection by *Mycobacterium avium* subsp. *Paratuberculosis* (Shandilya et al., 2023). In addition, gga-miR-181b-5p was reported to participate in host immune and inflammatory responses against infection of chicken macrophages by avian pathogenic *Escherichia coli* via activation of the TGF-β signaling pathway (Yang et al., 2023). However, further clarification of the changes to the miRNA expression profiles of host cells in response to *K*. *variicola* infection is needed to advance current knowledge of the mechanisms underlying resistance and susceptibility to bacterial infection.

The degree of activation of the host immune response is largely determined by the virulence of the pathogen and the spleen can elicit prompt innate and adaptive immune responses upon recognition of the specific antigen in the lymph and blood (Bronte and Pittet, 2023; Hosseindoust et al., 2023). However, due to the under developed nature of the lymphatic vessels and nodes of avian species, the spleen serves as the principal lymphatic organ, thereby acquiring a more significant role in immune function as compared to mammals via integration of the innate and adaptive immune responses involving the generation, maturation, and storage of lymphocytes (Smith and Hunt, 2004; Tiron and Vasilescu, 2008). Given the pivotal role of the spleen in the systemic immune response, it would be advantageous to identify genes activated in response to *K*. *variicola* infection and pathology.

Therefore, the aims of the present study were to characterize the miRNA expression profile of the chicken spleen in response to *K*. *variicola* infection and further identify related miRNA-mRNA regulatory networks. The results of this study will help to further clarify the pathogenesis of *K. variicola* in chickens and other avian species.

## Materials and methods

### Study approval

All animal experiments were approved by the Institutional Animal Care and Use Committee of the Institute of Animal Husbandry and Veterinary Science of Anhui Academy of Agricultural Sciences (permit no: AAAS-IAHVS-Po-2022-0051) and conducted in strict compliance with the guidelines of “Animal Research: Reporting of *In Vivo* Experiments” (Kilkenny et al., 2010).

### Bacterial strains and growth conditions

*K. variicola* strain AHKV-S01 (GenBank accession number: CP047360) was isolated from diseased chickens in China and verified by whole-genome sequencing. Two days prior to bacterial challenge, cultured *K. variicola* cells were streaked onto Luria–Bertani (LB) agar and incubated overnight at 37°C. Afterward, individual colonies of *K. variicola* were further cultured in 10 mL of LB broth overnight at 37°C with shaking. On the day of challenge, the bacteria were centrifuged at 5000 × g for 15 min and the resulting bacterial pellet was washed three times with phosphate-buffered saline (PBS) and resuspended in PBS. The bacteria in suspension were quantified with aspectrometer at a wavelength of 600 nm. Finally, the inoculum was adjusted with PBS to the desired bacterial concentration of 10^8^ CFU/mL (Shen et al., 2021).

### Animal study

Embryonated eggs from specific pathogen-free (SPF) Leghorn chickens were purchased from Beijing Merial Vital Laboratory Animal Technology Co. Ltd. (Beijing, China). The SPF eggs were hatched under a controlled environment and the chicks were raised in negative-pressure isolators to protect against all pathogens. At the age of 7 days, 30 SPF chickens were orally challenged with 10^8^ CFU of *K. variicola* (1 mL) and 15 others received 1 mL of PBS by the same method as a control group (Yin et al., 2023). At 24 h post infection, the chickens were euthanized and the spleens were immediately harvested, frozen in liquid nitrogen, and stored at -80°C. For histopathological analysis, the spleens of chickens from the infected and control groups were fixed with 4% paraformaldehyde for 48 h, then dehydrated, embedded in paraffin, and cut into 4-μm-thick sections, which were stained with hematoxylin and eosin as previously described (Wang et al., 2017).

### Cell culture

DF-1 chicken fibroblasts were cultured in Dulbecco’s modified Eagle’s medium supplemented with 10% fetal bovine serum (Gibco™; Invitrogen Corporation, Carlsbad, CA, USA). Chicken HD11 macrophages were cultured in Roswell Park Memorial Institute 1640 medium containing 20 mM L-glutamine (Gibco™; Invitrogen Corporation) and 10% fetal bovine serum (Gibco™; Invitrogen Corporation). All cells were incubated at 37°C under an atmosphere of 5% CO_2_ (Yang et al., 2023).

### MiRNA sequencing and bioinformatics analysis of target genes

MiRNA profiling of the spleens of *K. variicola*-infected chickens was performed by Shanghai OE Biotech Co., Ltd. (Shanghai, China; http://www.oebiotech.com). Briefly, RNA was extracted, labeled, and hybridized to a rat miRNA microarray (070154 R; V21.0 8 × 15K; Agilent Technologies, Inc., Santa Clara, CA, USA). GeneSpring software (v13.1; Agilent Technologies, Inc.) was used to normalize the raw data for identification of differentially expressed miRNAs (DEmiRNAs), which were defined as a fold change ≥1.5 and probability (P) value ≤ 0.05. Target genes of DEmiRNAs were identified in reference to the databases TargetScan (https://www.targetscan.org/vert_80/) and microRNAorg (https://ngdc.cncb.ac.cn/) (Griffiths-Jones et al., 2008). Functional and pathway enrichment analyses of all putative genes were performed in reference to the Gene Ontology (GO; https://geneontology.org/) and Kyoto Encyclopedia of Genes and Genomes (KEGG; https://www.genome.jp/kegg/) databases based on a threshold of significance of *P* ≤ 0.05 (Ye et al., 2018; Pian et al., 2020). Cytoscape software (http://www.cytoscape.org/) was used to identify potential regulatory relationships between the DEmiRNAs and target genes (Ragueneau et al., 2021).

### Integration analysis of the miRNA and mRNA data

Two datasets of miRNA and transcriptome sequences were used for integration analysis between the DEmiRNAs and mRNAs. Pearson correlation coefficients (r values) between the DEmiRNAs and related target genes were calculated using Excel software (Microsoft Corporation, Redmond, WA, USA). Strong positive and negative correlations between the DEmiRNAs and mRNAs were defined as *r* values > 0.8 or < -0.8, respectively (Jia et al., 2017).

### Plasmid construction and cell transfection

HD11 macrophages were transfected with mimic miRNAs and negative control ligonucleotides (Shanghai GenePharma Co., Ltd., Shanghai, China) using Lipofectamine 2000 transfection reagent (Invitrogen Corporation) in accordance with the manufacturer’s instructions. After 36 h of transfection, total RNA was isolated using TRIzol reagent (Invitrogen Corporation).

### Dual luciferase reporter assay

Target sites of gga-miR-2954 were validated using pmirGLO-basic vectors (Wuhan Gene Create Biological Engineering Co., Ltd., Wuhan, China) with double-luciferase reporter genes. Fragments of the 3’ untranslated regions (3′-UTRs) containing potential binding sites of *E2F2*, *STAB1*, and *DOK2* were cloned from chicken DNA samples. DF-1 cells were transfected with either 500 ng of the wild-type or mutant vector and 500 ng of the gga-miR-2954 mimic for 48 h using Lipofectamine 2000 transfection reagent (Invitrogen Corporation). Then, the cells were collected and successful transfection was validated using a Dual Luciferase Reporter Assay Kit (Promega Corporation, Madison, WI, USA) in accordance with the manufacturer’s instructions. Each experiment was repeated three times (Sheedy et al., 2010).

### Real-time quantitative polymerase chain reaction

Total RNA was polyadenylated with poly-A polymerase and reverse transcribed into complementary DNA using a poly (T) adapter primer (Sangon, Shanghai, China). RT-qPCR was performed with a StepOne™ Real-Time PCR System (Applied Biosystems, Carlsbad, CA, USA), a miRcute Plus miRNA qPCR Kit (SYBR Green) (Tiangen Biotech (Beijing) Co., Ltd., Beijing, China), SYBR ^®^ Green Realtime PCR Master Mix (Toyobo Co., Ltd., Osaka, Japan), and the primers listed in **Supplementary Material** in **Supplementary Table S1 and S2** to measure the mRNA and miRNA expression levels, respectively. Three independent biological replicates were assessed for each gene. The relative expression levels of the miRNAs and mRNAs were calculated with the 2^−ΔΔct^ method against GAPDH and U6 as reference genes (Livak and Schmittgen, 2001).

### Statistical analysis

All statistical analyses (Student’s t-test) were conducted with Prism 5 software (GraphPad Software, Inc., San Diego, CA, USA). A *P* value < 0.05 was considered statistically significant.

### Data availability

The chicken spleen mRNA and miRNA reads were deposited in the GenBank database (https://www.ncbi.nlm.nih.gov/) under the accession numbers PRJNA988896 and PRJNA1006787, respectively.

## Results

### Anatomical and histological changes to chicken spleens infected with *K. variicola*

**Figure 1:**
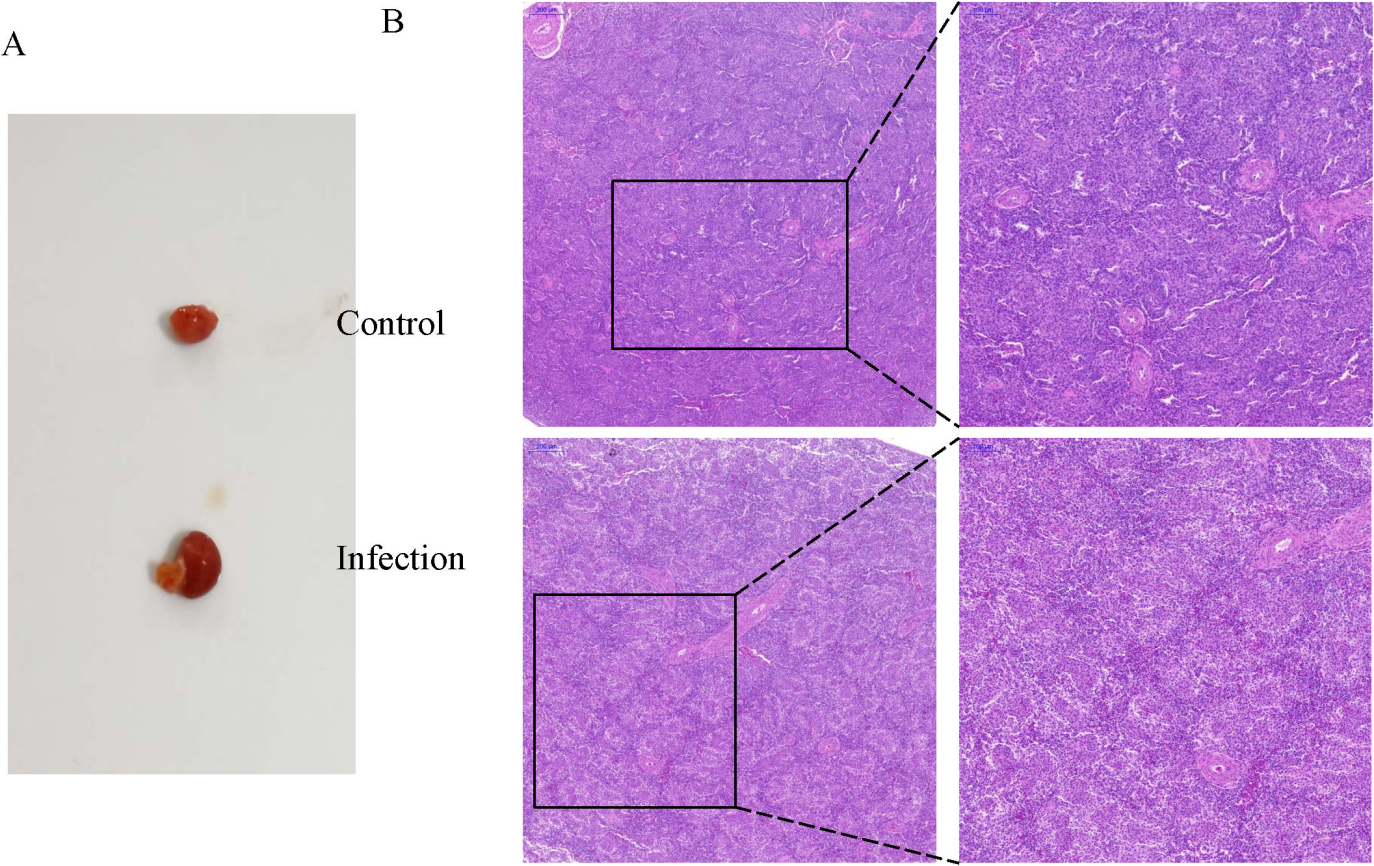
Changes in the chicken spleens after *K variicola* infection. **(A)** Morphological characteristics of the spleens of healthy and infected chickens. **(B)** Histopathological changes of the chicken spleens in the control and infected group.

Anatomical analysis revealed that *K. variicola* infection caused significant enlargement of the spleen (**Figure 1A**), while histological analysis revealed severe depletion of lymphoid cells in the follicles and infiltration of inflammatory cells (**Figure 1B**).

### Quality assessment of total miRNA sequencing data

Total RNA from six samples (control 1, control 2, control 3, infection1, infection 2, and infection 3) was isolated and sequenced. Nearly 20.55–25.23 megabytes (MB) of raw reads were generated, of which 20.9–24.05 MB of clean reads were obtained by removing alllow-quality reads, sequences containing unknown bases (N), and questionable Q20 values and lengths. In addition, 0.58–1.01 MB of unique identifier reads were obtained (**Table 1**). After deduplication control, 19,516,698–24,047,450 reads were obtained, of which 94.81% (average, 20,594,524) were validated and successfully mapped to the *Gallus gallus* reference genome (**Table 2**).

**Table 1:** Summary of the miRNA-seq data.

Sample	RawReads	Reads_trimmed_length	Reads_ trimmed_Q20	Reads_ trimmed_N	CleanReads	CleanReads_uniq
Control 1	25.23M	24.84M	24.05M	24.05M	24.05M	0.69M
Control 2	22.7M	22.41M	21.68M	21.68M	21.68M	1.01M
Control 3	23.29M	23.01M	22.29M	22.28M	22.28M	0.58M
Infection 1	20.55M	20.29M	19.52M	19.52M	19.52M	0.77M
Infection 2	21.15M	20.87M	20.1M	20.09M	20.09M	0.72M
Infection 3	23.46M	23.14M	22.4M	22.4M	22.4M	1.01M

**Table 2:** The mapping information of miRNA-seq data.

Sample	Reads	Uniq reads	Aligned_reads	Aligned Uniq reads	Aligned(%)
Control 1	24,047,450	687,994	23,131,002	559,502	96.19%
Control 2	21,676,930	1,010,518	20,861,203	848,381	96.24%
Control 3	22,282,778	578,416	21,517,472	480,049	96.57%
Infection 1	19,516,698	773,057	17,943,816	596,024	91.94%
Infection 2	20,092,226	719,113	18,631,000	578,359	92.73%
Infection 3	22,399,402	1,009,914	21,482,654	855,707	95.91%

### Identification and characterization of miRNAs

**Figure 2:**
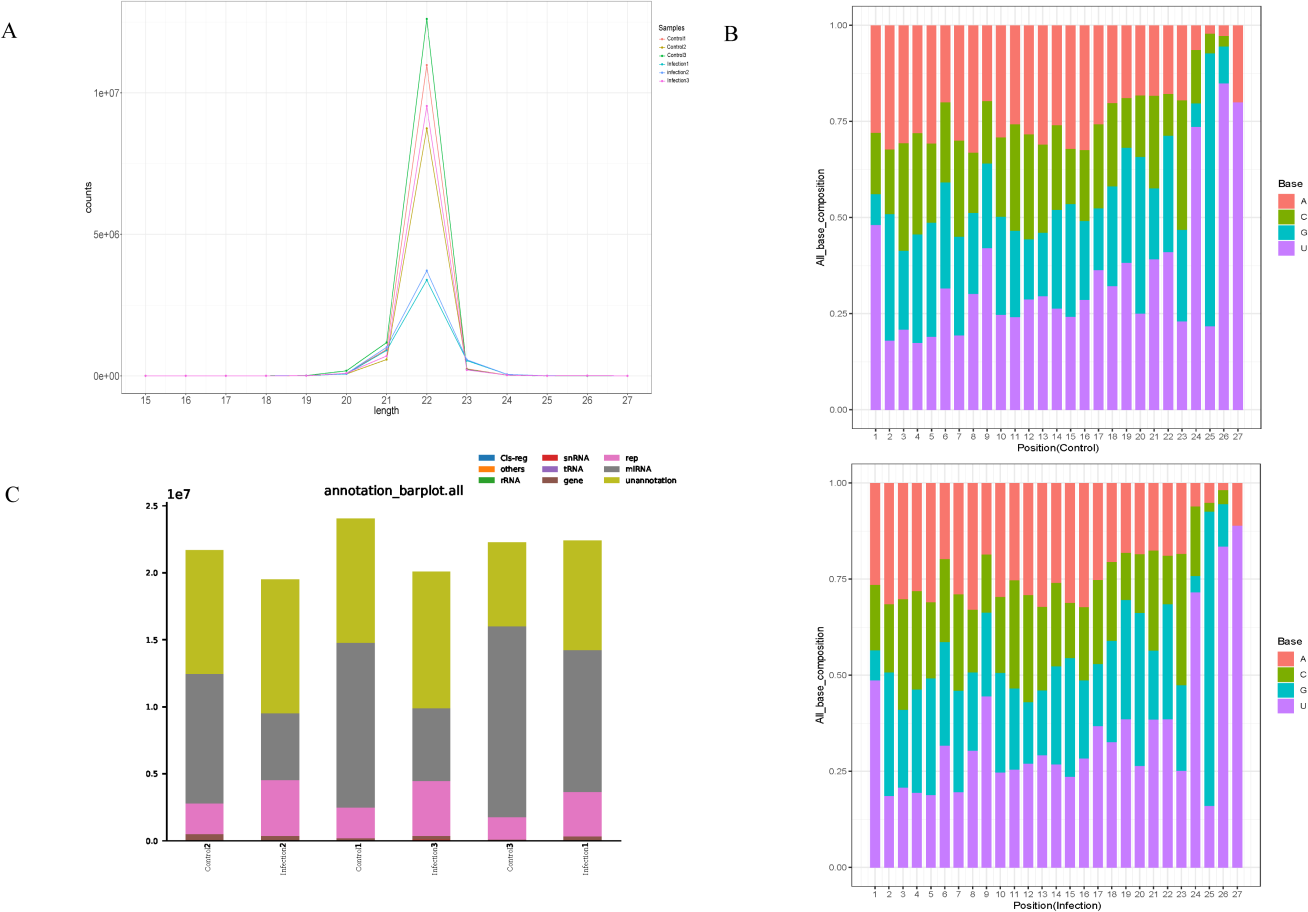
Characteristics of small RNA in the control and infected group. **(A)** Length distribution of the clean reads of the sequences. **(B)** Size range and base bias at the first position of miRNAs identified in control and infected group. **(C)** The different RNA categories of the miRNA-seq data.

The lengths of the six libraries varied from 15 to 27 nucleotides (**Figure 2A**). Most of the miRNAs of the libraries were 22 nucleotides in length and the first nucleotide of the identified miRNAs exhibited a strong preference for ′U′ at the 5′-end (**Figure 2B**). In addition, the final small RNAs were annotated based on the RNA families (Rfam) database, and classified as miRNA, rRNA, snRNA, tRNA, Cis-region, repeat, other Rfam-RNA, and unannotation (**Figure 2C**). Various miRNAs were enriched in the chicken spleen in response to *K. variicola* infection.

### Identification of DEmiRNAs

**Figure 3:**
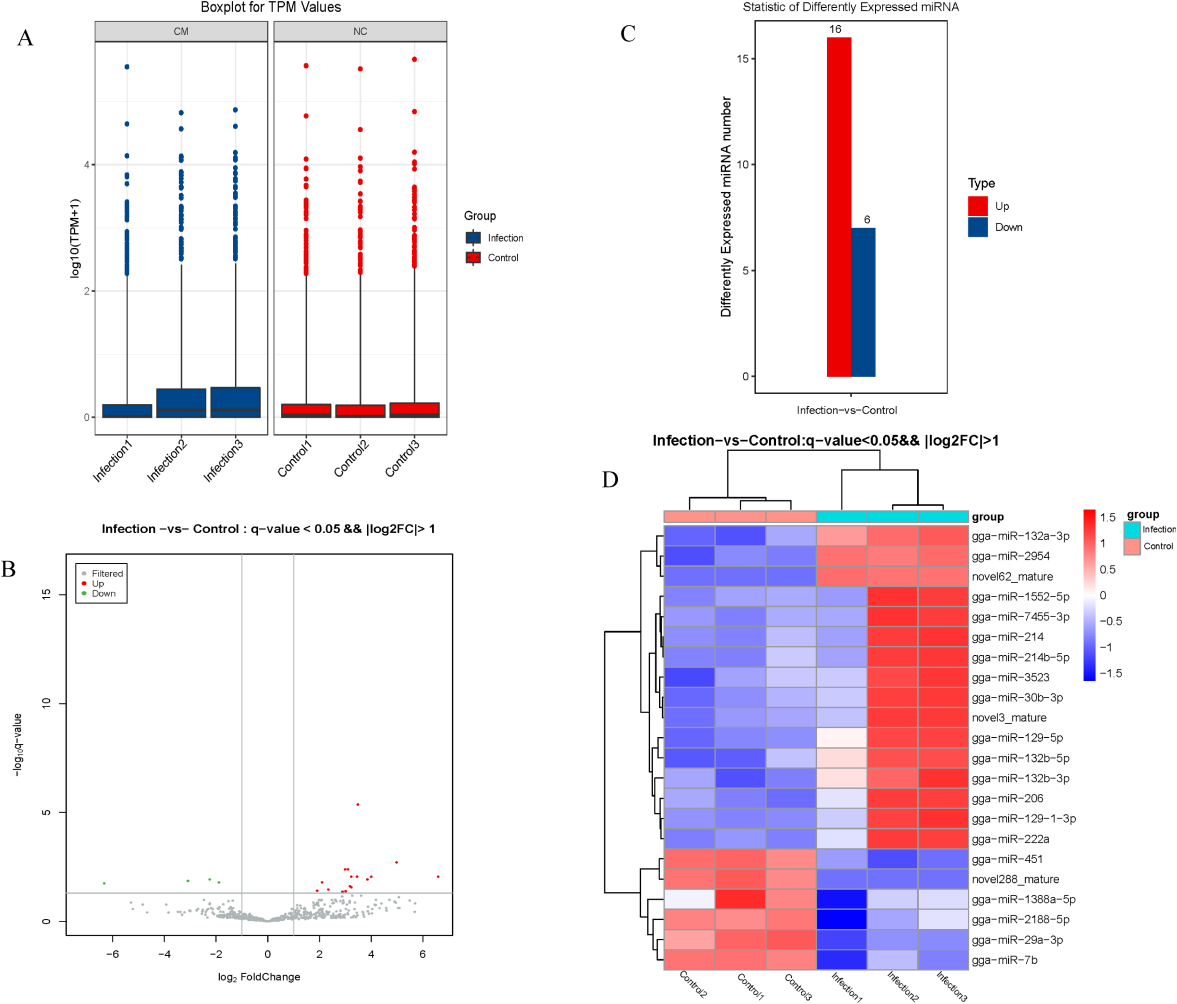
Analysis of differentially expressed miRNA. **(A)** The expression patterns of total genes in different samples. **(B)** Volcano plot map of differentially expressed miRNA. **(C)** The numbers of up- and down-regulated differentially expressed miRNAs **(D)** Heat map of differentially expressed miRNA.

A box-whisker plot (**Figure 3A**) based on normalized expression of miRNAs in each sample and a volcano plot showing distinct differences in the miRNA profiles of the control and infection groups (**Figure 3B**) were generated. Using *q*< 0.05 and |log2 (expression level)|>1 as the cut off values, 22 DEmiRNAs (16 up-regulated and 6 down-regulated) were identified between the control and infection groups (**Figures 3C, D**).

### Functional annotation of potential target genes of the DEmiRNAs

**Figure 4:**
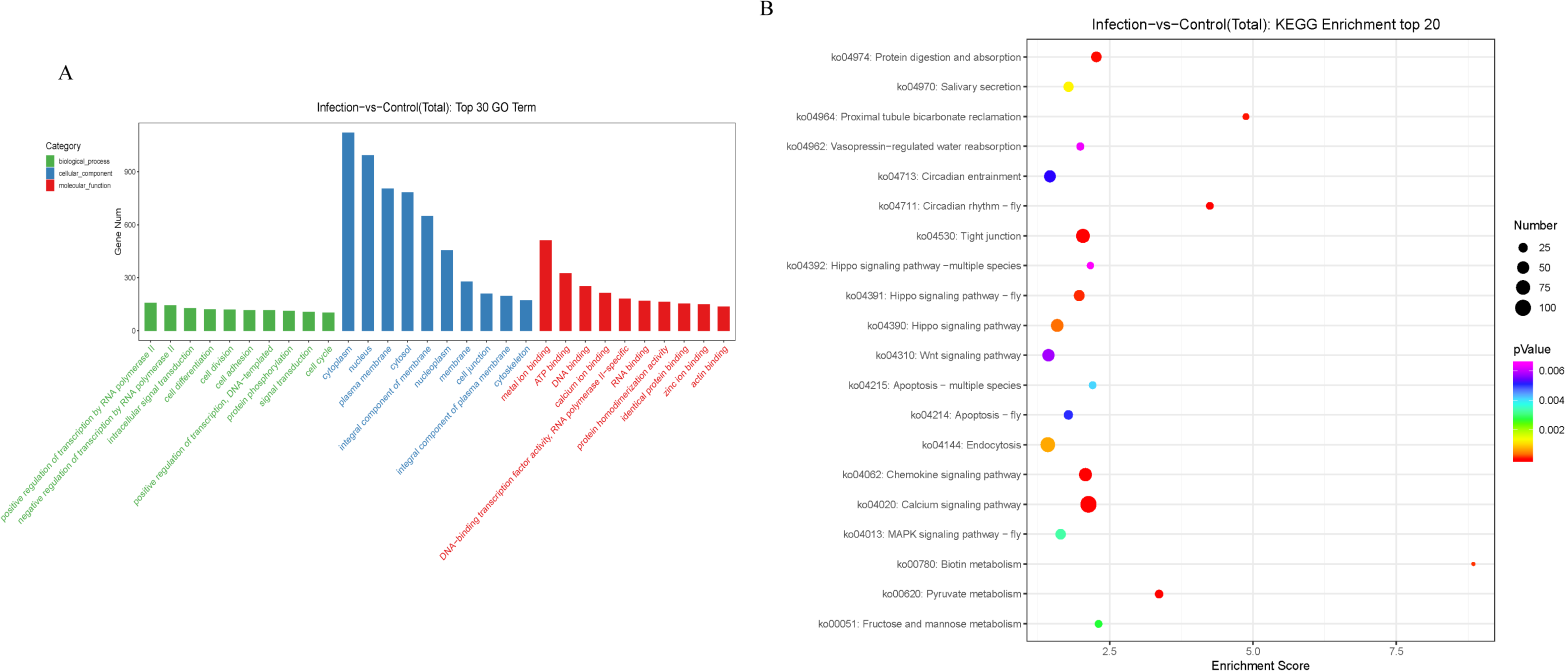
GO and KEGG analysis of potential target genes of differentially expressed miRNAs. **(A)** The GO terms ranked by the fold enrichment and enrichment score are shown. The terms related to biological process (BP), cellular component (CC), and molecular function (MF) are represented by green, blue, and red, respectively. **(B)** The identified KEGG pathways ranked by the *P*-value are shown. The color of the circle represents the adjusted P-value for each pathway. The size of the circle represents the number of genes enriched in each pathway.

Functional annotation and pathway enrichment analyses of the potential target genes of the identified miRNAs were performed. The 10 most significantly enriched GO terms (biological processes, cellular components, and molecular functions) of the putative target genes of the DEmiRNAs between the infection and control groups are presented in **Figure 4A**. The biological processes were mainly involved in regulation of transcription by RNA polymerase II, intracellular signal transduction, cell differentiation, cell division, cell adhesion, DNA-templated, protein phosphorylation, and the cell cycle, while the cellular components were mainly involved in cytoplasm, nucleus, plasma membrane, cytosol, integral component of membrane, nucleoplasm, membrane, cell junction, integral component of plasma membrane, and cytoskeleton, and the molecular functions mainly involved metal ion binding, ATP binding, DNA binding, calcium ion binding, DNA-binding transcription factor activity, RNA polymerase II-specific, RNA binding, protein homodimerization activity, identical protein binding, zinc ion binding, and actin binding.

KEGG pathway analysis demonstrated that the mRNAs targeted by the DEmiRNAs were primarily enriched in metabolism and immune signaling pathways (**Figure 4B**).

### The miRNA-mRNA regulatory relationships in the chicken spleen after *K. variicola* infection

**Figure 5:**
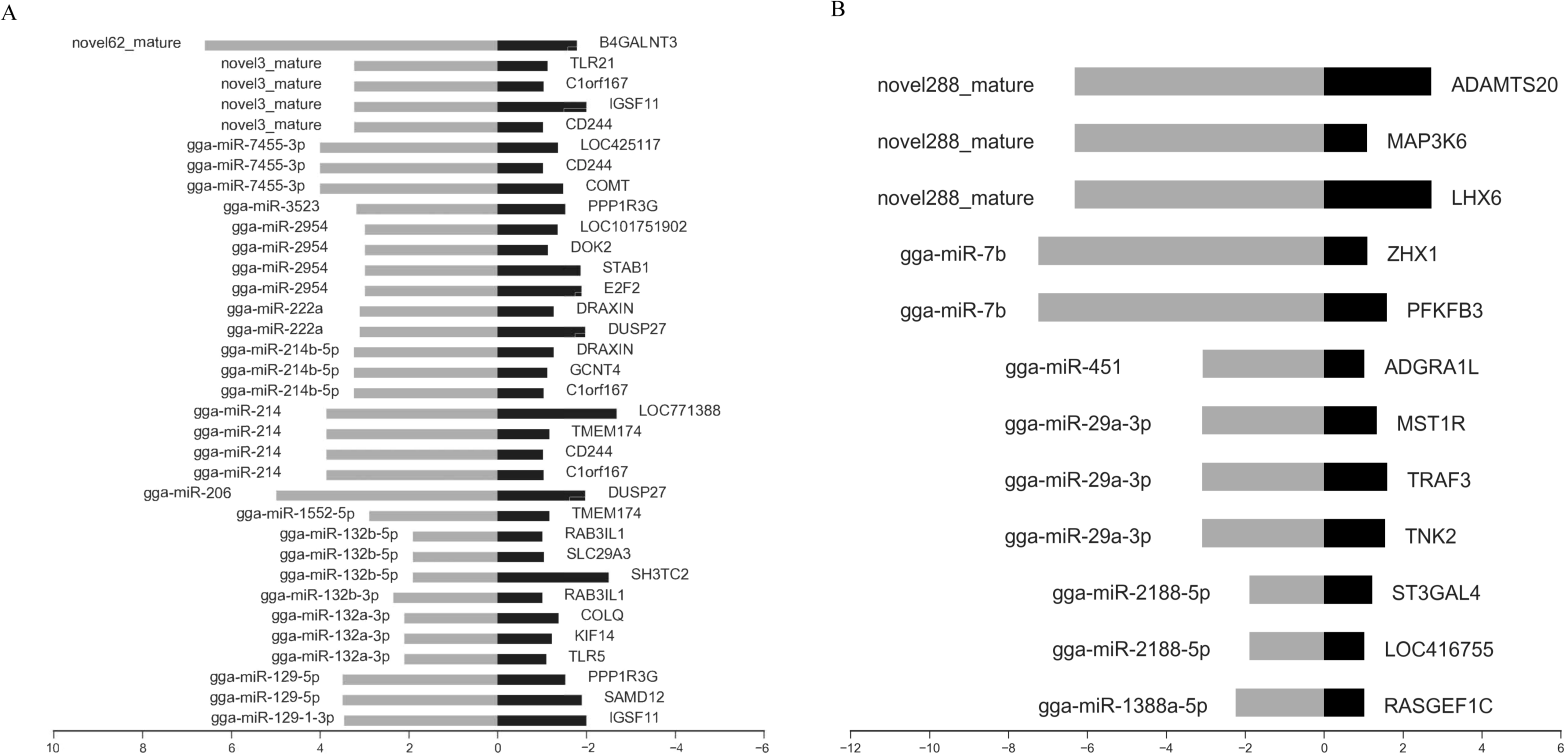
Integrated analysis of differently expressed miRNAs and mRNAs during *K variicola* infection. **(A)** Negatively correlated expression of upregulated miRNAs and predicted targets. **(B)** Negatively correlated expression of downregulated miRNAs and predicted targets. The horizontal axis indicates the miRNA and mRNA log2 ratio value of the infection versus control groups, and the vertical axis indicates inverse expression pairs between miRNAs and mRNAs. The Pearson correlation coefficient was used to estimate the expression relationships of miRNAs and mRNAs. Only miRNA-mRNA pairs with an *r* of less than -0.8 were considered to be strongly inversely correlated.

Integrated miRNA and mRNA expression approaches were performed by pairwise correlation coefficient analysis to construct miRNA-mRNA regulatory networks. With a threshold of *r* = -0.80, 45 potential miRNA-mRNA pairs were identified, which included 34 pairs with 16 upregulated miRNAs and 27 genes (**Figure 5A**) and 12 pairs with 6 downregulated miRNAs and 12 genes (**Figure 5B**).

### Validation of miRNA-seq and RNA-seq data by RT-qPCR

**Figure 6:**
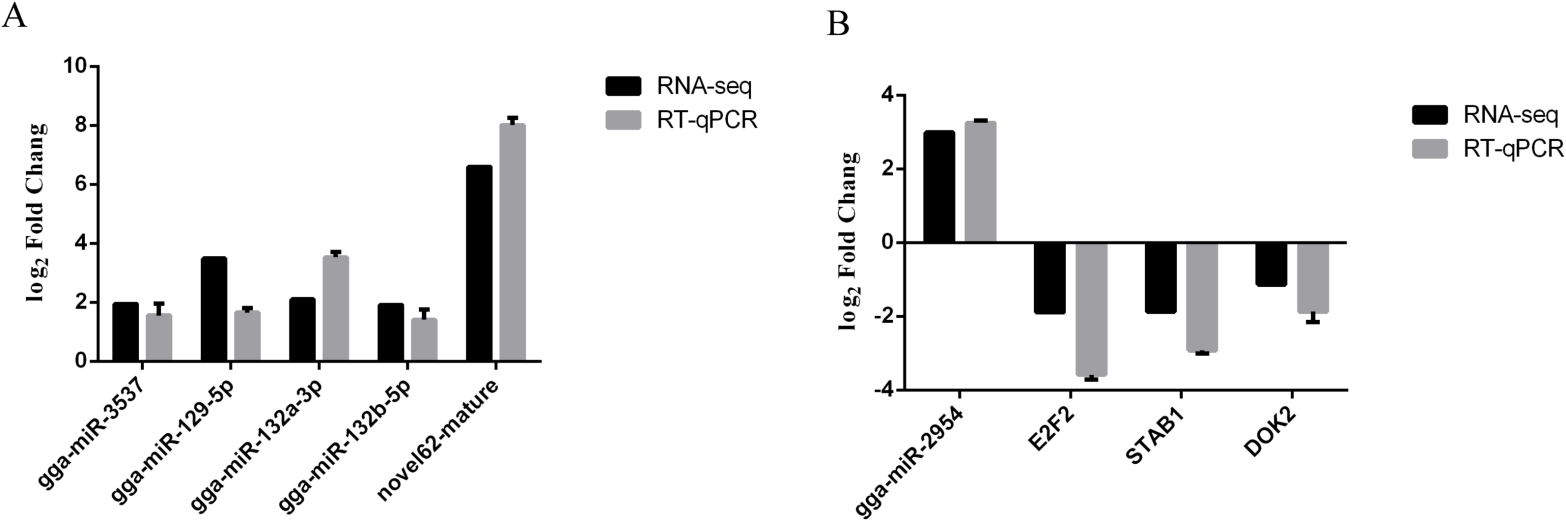
RT-qPCR detection of differentially expressed miRNA-mRNA pairs among the control and infected group. **(A)** Validation of miRNA-seq data by RT-qPCR. **(B)** gga-miR-2954 versus target genes. Relative expression level of each mRNAs and miRNAs were calculated using the 2^−ΔΔCt^ method. Values represent mean ± SEM from three independent experiments.

The expression levels of five differentially expressed miRNAs were determined with RT–qPCR to check the reliability of our miRNA-seq data. The results showed that the trends in these differentially expressed miRNAs determined with miRNA-seq were consistent with those determined with RT–qPCR (**Figure 6A**). In addition, the sequencing results revealed that gga-miR-2954 expression was significantly increased in the infection group as compared to the control group and the potential target genes included *E2F2*, *STAB1*, and *DOK2*. To validate the reliability of the miRNA and mRNA expression profiles obtained from the sequencing data, specific miRNA-mRNA pairs were selected for RT-qPCR analysis. The results demonstrated that the expression levels of *E2F2*, *STAB1*, and *DOK2* were significantly decreased following *K. variicola* infection, while gga-miR-2954 expression was significantly increased (**Figure 6B**). These results revealed that expression of gga-miR-2954 was negatively correlated to expression of *E2F2*, *STAB1*, and *DOK2* after *K. variicola* infection.

### Validation of gga-miR-2954 target genes

**Figure 7:**
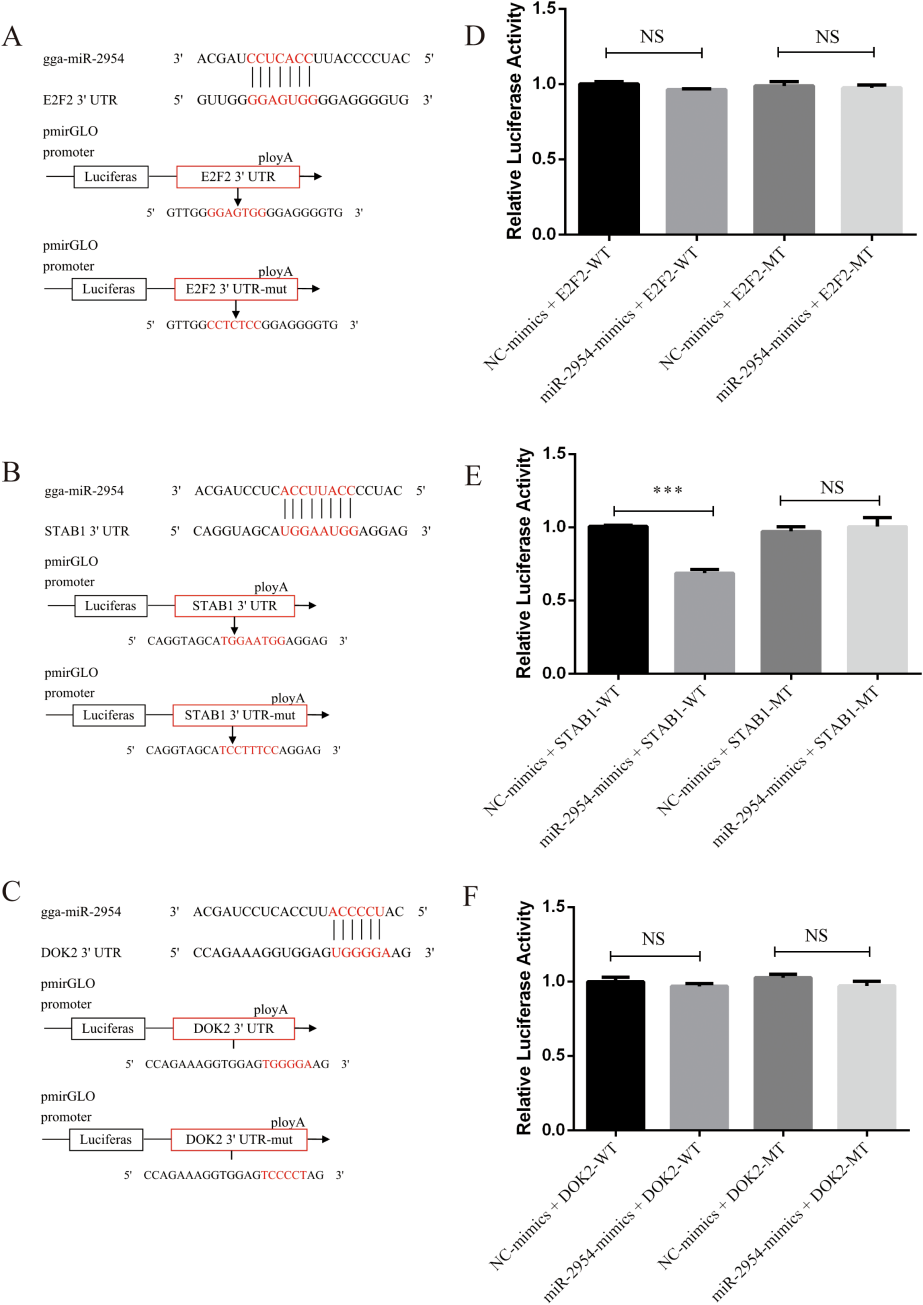
Validation of potential targets downregulated by gga-miR-2954 in DF-1 cell lines. **(A)** The binding site of gga-miR-2954 and the 3’ UTR of *E2F2*. **(B)** The binding site of gga-miR-2954 and the 3’ UTR of *STAB1*. **(C)** The binding site of gga-miR-2954 and the 3’ UTR of *DOK2*. **(D)** Validation of the targeting relationships between gga-miR-2954 and *E2F2* by using dual luciferase reporter assay. **(E)** Validation of the targeting relationships between gga-miR-2954 and *STAB1* by using dual luciferase reporter assay. **(F)** Validation of the targeting relationships between gga-miR-2954 and *DOK2* by using dual luciferase reporter assay. ***P<0.001, NS: P>0.05.

The dual-luciferase assay was employed to confirm the ability of gga-miR-2954 to pair with the 3′-UTR of the potential target genes *E2F2*, *STAB1*, and *DOK2* during *K. variicola* infection. The wild-type and mutant 3′-UTR sequences of *E2F2*, *STAB1*, and *DOK2* were each inserted into pmirGLO vectors (**Figures 7A–C**). Relative luciferase activity of DF-1 cells co-transfected with a gga-miR-2954 mimic or negative control and pmirGLO vectors containing the 3′-UTR of *STAB1* was significantly decreased, but not those co-transfected with the 3′-UTR of *E2F2* and *DOK2*, suggesting that gga-miR-2954 mediated transcription of *STAB1* by directly targeting the 3′-UTR (**Figures 7D–F**).

### Overexpression of gga-miR-2954 in chicken HD11 macrophages

**Figure 8:**
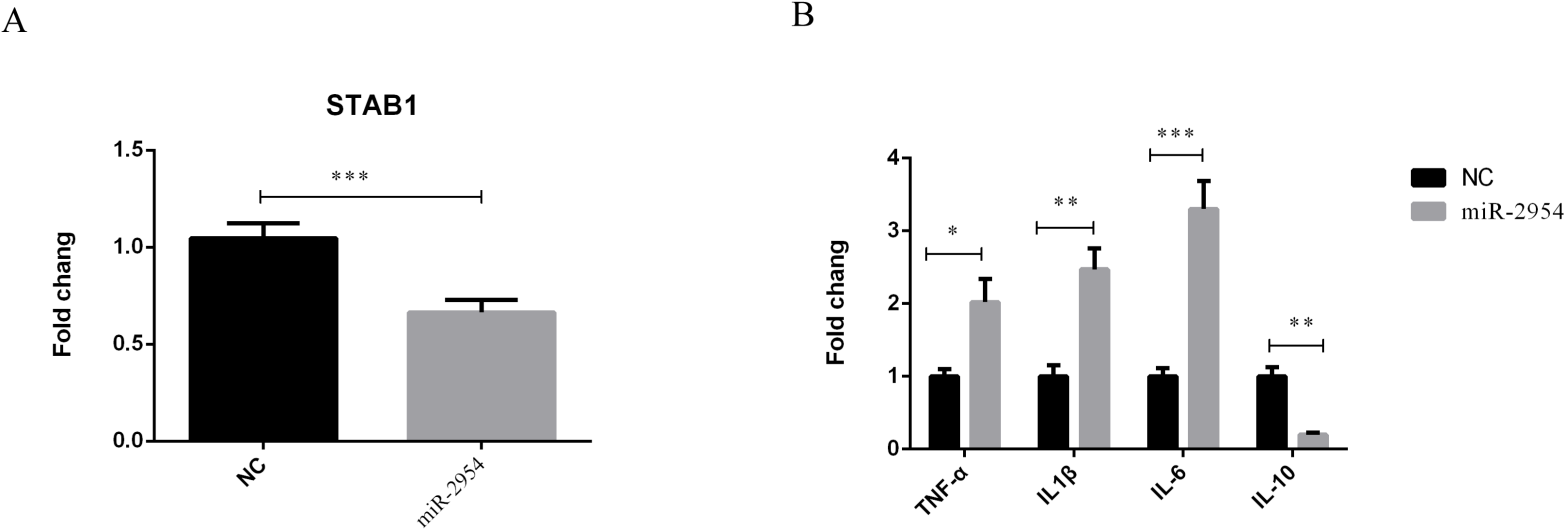
Validation of the relationship between *STAB1* and gga-miR-2954. **(A)** The mRNA expression level of *STAB1* in chicken macrophages transfected with gga-miR-2954 mimic. **(B)** Overexpression of gga-miR-2954 regulates mRNA expression levels of different cytokines. The expression levels of four pro-inflammatory mediators: TNF-α, IL1β, IL-6 and IL-10 were analysed in different groups by using RT-qPCR. *P<0.05, **P<0.01, ***P<0.001.

An HD11 macrophage-like cell line derived from bone marrow cells was used to further illustrate the association of gga-miR-2954 with *STAB1 in vitro*. After treatment with mimic miRNA, elevated expression of gga-miR-2954 significantly repressed mRNA expression levels of *STAB1* as compared to the negative control group (**Figure 8A**). Further investigation of the effects of gga-miR-2954 on the expression levels of different inflammatory cytokines showed that transfection with the gga-miR-2954 mimic suppressed expression of the anti-inflammatory cytokine IL-10 and enhanced expression of the pro-inflammatory cytokines TNF-α, IL1β and IL-6, as compared to the mimic negative control (**Figure 8B**). These results indicate that gga-miR-2954 plays a role in coordinated cytokine production elicited against *K. variicola* infection.

## Discussion

The bacterial pathogen *K. variicola* has become a threat to both human and animal health (Giannattasio-Ferraz et al., 2022). However, the responses of host cells to *K. variicola* infection remain unclear. Mounting evidence indicates that miRNAs may play critical roles in bacterial infection and the host immune response by regulating various target genes (Kimura et al., 2023). Therefore, the aim of the present study was to identify and characterize key miRNAs involved in the immune response of chickens infected with *K. variicola* in order to provide new ideas for prevention and control.

The miRNA expression profiling data led to the identification of 22 DEmiRNAs and the functions of the associated target genes were predicted by GO term and KEGG pathway enrichment analyses. The results of GO enrichment analysis showed that some functions related to cell structure changed after infection by *K. variicola*. The effector proteins and toxins of pathogenic bacteria influence the cytoskeleton of the host cell. The structural proteins of the cytoskeleton protect the host cell against pathogen-induced damage by signal recognition or transmission (Zhang et al., 2023). Thus, the cytoskeleton plays a crucial role in cell-autonomous immunity. KEGG pathway analysis showed that various miRNAs are involved in the regulatory mechanisms of host immunity in response to *K. variicola* infection. In the chicken spleen, relevant signaling pathways are rapidly triggered upon stimulation by an external pathogen. In the present study, the target genes of DEmiRNAs were involved in regulation of the MAPK, Wnt, and Hippo signaling pathways, as well as tight junction proteins and metabolism of pyruvate, fructose, and mannose. These signaling pathways participate in inflammation (Kim et al., 2022; Zong et al., 2023; Jiang et al., 2024; Lei et al., 2024; St Louis et al., 2024), apoptosis (Zong et al., 2019; Wang et al., 2023; Zhao et al., 2024; Zhuang et al., 2024), and immune defenses (Ren et al., 2022; Huang and Huang, 2023; Tang et al., 2023; Vu et al., 2023). Collectively, these findings suggest that *K. variicola* mediates host responses via DEmiRNAs.

Previously reported RNA-seq transcriptome profiles were referenced to identify miRNAs related to host resistance (Jia et al., 2017). Interestingly, gga-miR-2954 and *STAB1* were the most significant miRNA–mRNA pair. Prior studies have established that gga-miR-2954 plays important roles in various diseases of chickens. For example, gga-miR-2954 was reportedly associated with the immune response of chicken kidneys to the infectious bronchitis virus (Yang et al., 2017). Similarly, gga-miR-2954 expression was highly upregulated in chicken embryo fibroblasts infected with the reticuloendotheliosis virus (Yu et al., 2017). In the present study, gga-miR-2954 was associated with production of inflammatory cytokines by targeting *STAB1* during *K. variicola* infection. STAB1 is a highly conserved type I transmembrane protein mainly expressed in sinusoidal endothelial cells of the spleen and liver (Goerdt et al., 1991). It has been demonstrated that in *L. monocytogenes*-infected murine macrophages and endothelial cells, reduced *STAB1* expression enhances bacterial uptake by recognising surface components of the pathogen, thereby increasing macrophage phagocytic activity (Pombinho et al., 2021). STAB1 deficiency triggers excessive release of pro-inflammatory cytokines (e.g. IL-6, TNF-α), shifting macrophage polarisation from an anti-inflammatory (M2) to a pro-inflammatory (M1) phenotype. This imbalance exacerbates tissue damage and disrupts immune homeostasis (Rantakari et al., 2016). In the present study, gga-miR-2954 expression was significantly upregulated in the *K. variicola*-infected group as compared to the non-infected control group and might have enhanced inflammation by inhibiting expression of *STAB1*. Collectively, these data suggest that the DEmiRNAs, especially gga-miR-2954, play important roles in host immune and inflammatory responses against *K. variicola* infection.

In conclusions, This study characterized the miRNA expression profile of the chicken spleen in response to *K. variicola* infection. In total, 22 DE miRNAs were identified between the infected and control groups. Integration analysis of DEmiRNAs and mRNA found that gga-miR-2954 plays an important role in *K. variicola* infection. Specifically, gga-miR-2954 directly targeted *STAB1* to further modulate expression of inflammatory cytokines. These findings will help to clarify the miRNA expression profile of the chicken spleen in response to *K. variicola* infection, provide information about potential vaccine targets, and assist genetic selection for resistance to *K. variicola*.

## Data Availability

The datasets presented in this study can be found in online repositories. The names of the repository/repositories and accession number(s) can be found in the article/**Supplementary Material**.
