# Supplementary material for: MicroRNA warfare: how chickens combat Klebsiella variicola with gga-miR-2954
Source: Front Cell Infect Microbiol. 2025 May 8;15:1544506. doi: 10.3389/fcimb.2025.1544506 (PMC12095194; doi:10.3389/fcimb.2025.1544506)
Supplement: Supplementary file 1 [file Table1.docx]

**Table S1.** **Sequences of primers used in this study**

| **Gene ID** | **Primer name** | **Primer Sequence (5’-3’)** | **Product length(bp)** |
| --- | --- | --- | --- |
| XM_040651984 | E2F2-F  E2F2-R | GCTGAGACGGAGGCAACTTT  TTGCCCATGGCTCTGTTTGA | 98 |
| XM_046900193 | STAB1-F  STAB1-R | TACACTGGCCCCAGATGTGA  ATTGCAGTCCCGTCCTTGAC | 95 |
| XM_003642549 | DOK2-F  DOK2-R | GCGCCAAGAGCATCTACAC  AGGGAACGGCGTACTCCAAT | 126 |
| XM_430147.5 | TNFα-F  TNFα-R | CTGCTTGCCTGCTTGATT  CTGCGATGCTTTGACCTG | 109 |
| XM_015297469.1 | IL1B-F  IL1B-R | CCTTCACCCTCAGCTTTCAC  GCCCTCCCATCCTTACCTT | 139 |
| XM_015281283.2 | IL6-F  IL6-R | AGTGAGGCAGTGGGTGAT  GCCAATACAGGTGGATAAG | 134 |
| XM_025143715 | IL10-F  IL10-R | AAAGATTTGATGGGTAGTGAG  ACGGGTGATATTAAGGGTAT | 124 |
| NM_204305 | GAPDH-F  GAPDH-R | CAGAACATCATCCCAGCGTC  GGCAGGTCAGGTCAACAAC | 133 |

**Table S2. Primers used to confirm miRNA expression with qRT-PCR**

| **miRNA Name** | **miRNA Sequence (5’-3')** | **RT Primer Sequence (5’-3’)** | **Forward PCR Primer Sequence (5’-3')** |
| --- | --- | --- | --- |
| gga-miR-3537 | GTGAGTGCTGTAGGATGGGGCT | GTCGTATCCAGTGCGTGTCGTGGAGTCGGCAATTGCACTGGATACGACGAGCCCC | ACGTGGTGAGTGCTGTAGGA |
| gga-miR-129-5p | CTTTTTGCGGTCTGGGCTTGC | GTCGTATCCAGTGCGTGTCGTGGAGTCGGCAATTGCACTGGATACGACGCAAGC | CTAGCCTTTTTGCGGTCTGG |
| gga-miR-132a-3p | TAACAGTCTACAGCCATGGTCG | GTCGTATCCAGTGCGTGTCGTGGAGTCGGCAATTGCACTGGATACGACCGACCA | CGGCATAACAGTCTACAGCCA |
| gga-miR-132b-5p | ACCATGGCTGTAGACTGTTAC | GTCGTATCCAGTGCGTGTCGTGGAGTCGGCAATTGCACTGGATACGACGTAACA | GCGTTACCATGGCTGTAGAC |
| gga-miR-2954 | CATCCCCATTCCACTCCTAGCA | GTCGTATCCAGTGCGTGTCGTGGAGTCGGCAATTGCACTGGATACGACTGCTAG | CGTACATCCCCATTCCACTC |
| novel62_mature | CTCGTGACGAGTGTGAGCCC | GTCGTATCCAGTGCGTGTCGTGGAGTCGGCAATTGCACTGGATACGACGGGCTC | CCTGAACTCGTGACGAGTGT |
| microRNA U6 |  | CTCGCTTCGGCAGCACA | AACGCTTCACGAATTTGCGT |
